# High-Dynamic-Range Tone Mapping in Intelligent Automotive Systems

**DOI:** 10.3390/s23125767

**Published:** 2023-06-20

**Authors:** Ivana Shopovska, Ana Stojkovic, Jan Aelterman, David Van Hamme, Wilfried Philips

**Affiliations:** IMEC-IPI-Ghent University, 9000 Ghent, Belgium; ana.stojkovic@ugent.be (A.S.); jan.aelterman@ugent.be (J.A.); david.vanhamme@ugent.be (D.V.H.); wilfried.philips@ugent.be (W.P.)

**Keywords:** high dynamic range, tone mapping, deep learning, object detection, autonomous driving

## Abstract

Intelligent driver assistance systems are becoming increasingly popular in modern passenger vehicles. A crucial component of intelligent vehicles is the ability to detect vulnerable road users (VRUs) for an early and safe response. However, standard imaging sensors perform poorly in conditions of strong illumination contrast, such as approaching a tunnel or at night, due to their dynamic range limitations. In this paper, we focus on the use of high-dynamic-range (HDR) imaging sensors in vehicle perception systems and the subsequent need for tone mapping of the acquired data into a standard 8-bit representation. To our knowledge, no previous studies have evaluated the impact of tone mapping on object detection performance. We investigate the potential for optimizing HDR tone mapping to achieve a natural image appearance while facilitating object detection of state-of-the-art detectors designed for standard dynamic range (SDR) images. Our proposed approach relies on a lightweight convolutional neural network (CNN) that tone maps HDR video frames into a standard 8-bit representation. We introduce a novel training approach called detection-informed tone mapping (DI-TM) and evaluate its performance with respect to its effectiveness and robustness in various scene conditions, as well as its performance relative to an existing state-of-the-art tone mapping method. The results show that the proposed DI-TM method achieves the best results in terms of detection performance metrics in challenging dynamic range conditions, while both methods perform well in typical, non-challenging conditions. In challenging conditions, our method improves the detection F2 score by 13%. Compared to SDR images, the increase in $F_{2}$ score is 49%.

## 1. Introduction

According to studies by the Governors Highway Safety Association (GHSA) [1] and the National Highway Traffic Safety Administration (NHTSA) [2], between 50% and 75% of fatal traffic accidents involving pedestrians occur at night, despite the decreased traffic flow. One of the main contributing factors to nighttime accidents is impaired visibility due to insufficient light. Although the human eye is adaptable to a wide range of light conditions, it has trouble adjusting to low-light conditions. Blinding lights are also a significant challenge that impairs a driver’s vision.

Increasing the vehicle’s autonomy is expected to improve traffic safety by reducing the impact of human error [3,4]. The intermediate steps towards achieving full autonomy include the incorporation of increasingly intelligent sensing and control technologies in vehicles to assist human drivers [5,6], referred to as “advanced driver assistance systems” (ADAS).

In the context of challenging light conditions, the ratio between the brightest and the darkest perceivable light level is defined as the dynamic range (DR). Digital cameras used in commercial automotive vision systems are more limited in dynamic range than the eye, typically at around 70 dB. This is insufficient for a number of driving conditions. For example, oncoming car headlights at night or the sun low at the horizon are blinding to a typical sensor. Entering and exiting a tunnel introduces challenges of the stark contrast between dark and bright, where the sensitivity of a standard sensor can adapt well only to the bright or dark areas of the scene, but not both simultaneously. Such conditions impair visibility and pose a danger to drivers and other road users.

For ADAS and autonomous vehicles, using a high-dynamic-range (HDR) sensor with a low sensitivity threshold and a high saturation capacity is beneficial to increasing the signal-to-noise ratio (SNR) of the captured images. To encode HDR data, a representation of more than the standard 8 bits is required. For example, the automotive HDR sensor of the Sony IMX490 (Sony, Tokyo, Japan) uses 24 bits. However, processing high-bit-depth data requires more powerful computational resources than what is available on onboard computers. Another challenge is the limited availability of perception algorithms that work with high bit-depth data. The majority of state-of-the-art computer vision methods are optimized for 8-bit standard dynamic range (SDR) images, which makes them incompatible with high-bit-depth HDR data.

One solution is to adapt the existing algorithms to work directly with HDR input. The limitations of such a strategy are the need to redesign the state-of-the-art methods to a non-standard data representation, as well as the need to acquire sufficient new data to retrain the algorithms. We approach this problem from the perspective of adapting the input data instead, reasoning that it has a greater potential for facilitating the integration of HDR cameras in intelligent perception systems. Our approach is to non-linearly compress (tone map) the HDR data into a standard 8-bit representation, for application in object detection tasks for environment perception in traffic.

In this study, we demonstrated improved detection capabilities over a conventional tone-mapping-based detection system by specifically optimizing the tone mapping process for the detection of traffic-related objects. We compressed the intensity range of HDR images through object-aware tone mapping into an 8-bit/color channel representation while preserving the important image information. In comparison with other tone mapping operators developed for perceptual picture quality, our method helps to improve object detection while maintaining a natural image appearance.

Section 2 presents an overview of relevant literature related to high-dynamic-range imaging and tone mapping that optimizes the visual quality, as well as optimization of algorithms designed for more specific tasks that use HDR inputs. In Section 3, we describe the proposed algorithm for tone mapping using a deep neural net, optimized with respect to object detection performance for automotive applications. The experiments and results are presented in Section 4, and Section 5 concludes the paper and discusses future prospects.

## 2. Related Work

In the research literature, the vast majority of state-of-the-art tone mapping methods are optimized for enhancement of the image appearance for visualization purposes, based on models of the human visual system (HVS) or for subjective quality and artistic photography purposes [7,8,9,10,11,12,13,14,15,16,17]. The majority of these methods consider either local or global image features, but the most successful approaches typically analyze the input on multiple scales [9]. Global processing analyzes the overall illumination range, allowing preservation of the relative intensity differences without introducing artifacts such as halos and contrast reversal. A local analysis, on the other hand, helps preserve sharpness and enhance details. Nevertheless, ranking tone-mapping methods is not straightforward and depends on the evaluation criteria, as found by [11,12]. A recent method, iCAM06-m [10], builds upon the perceptual appearance model iCAM06 [8], and focuses on improving the color representation of tone-mapped HDR images. Subjective and objective evaluation experiments were carried out to evaluate the perceptual image quality of the results, achieving slightly better results for the modified model. However, these metrics do not adequately capture the effectiveness of the system for object detection purposes.

Another limitation of the aforementioned methods is that the performance is highly dependent on adequate hyperparameter tuning. To improve the robustness to changing conditions, more recent efforts rely on training a neural network for adapting to a broader range of scene contexts [14,15,16,17]. The limitations of these methods are that they are relatively computationally complex for implementation in real-time processing, as well as that they aim for general solutions with perceptual image quality goals. Furthermore, these goals are often subjective, without a precise and consistent performance metric.

To the knowledge of the authors, the quality of tone mapping operators has not been thoroughly evaluated with respect to object detection accuracy, and only a few studies exist that focus on object detection performance in HDR content [18,19,20]. Furthermore, the effectiveness of HDR imaging in various illumination conditions has not been extensively investigated in the automotive vision context. In this work, we aimed to optimize the process of tone mapping of HDR images to facilitate the detection of traffic-related objects in challenging light conditions.

In Ref. [18], the object detection performance was evaluated when seven different tone mapping operators (TMOs) were applied to HDR images. Moreover, the authors retrained three different neural-network-based object detector heads on a dataset of tone-mapped HDR images. The training dataset comprises a carefully selected subset of publicly available HDR images, manually annotated with labels for six object categories. The conclusions of this paper show that using tone-mapped HDR data results in better detection performance compared to using SDR data and that, in the majority of situations, most of the TM operators result in similar detection performance. However, the analysis does not differentiate between non-challenging and challenging light conditions, whereas the impact of different tone mapping methods is mostly observed in relatively few but safety-important challenging scenarios.

In continuation of this work, in [19], the authors explored the feasibility of training object detectors directly using HDR images. Due to the limited availability of HDR data for training, they created a pseudo-HDR dataset by applying a dynamic range expansion operator [21] on a set of SDR images. For evaluation, a small set of true HDR images was used. On average, the performance of detectors trained on HDR images was similar to that of detectors that were trained for SDR images and applied to tone-mapped HDR images. However, an isolated, small-scale study using 126 images of scenes with extreme light differences indicates a significant performance gain when HDR data are used, due to the better preservation of meaningful features.

Despite being evaluated on object detection performance, the aforementioned methods do not receive feedback from a task-specific module such as an object detector. A novel idea that combines multi-task learning is presented in [20]. The paper proposes a method for exposure selection as an alternative to using HDR sensors. A model that predicts optimal exposure values for image acquisition was trained in a joint end-to-end pipeline with image processing and object detection modules. The entire pipeline is supervised only with the object detector loss at the end. The reported results indicate that the proposed method outperforms the standard luminance-based and gradient-based auto-exposure control methods in supporting the object detection task for several automotive object categories.

In Ref. [22], the authors propose a novel approach, called TMO-Det, that jointly optimizes a generative adversarial network (GAN)-based tone mapping model and an object detector. Next to the discriminator, the GAN architecture is extended with a detection branch, which also provides feedback to the generator. The training loss function is augmented to enforce not only visual similarity to a classical tone mapping method but to also maximize object detection performance on the generated images. The main conclusions of this paper are that training the tone mapping GAN model jointly with a detector can achieve higher detection accuracy compared to classical TMOs as well as compared to GAN-based tone mapping architecture without jointly training with a detector. However, according to the authors, this increase was not very significant. Nonetheless, considering both detection performance measured by average precision (AP) and image quality measured by the Tone-Mapping Quality Index (TMQI) [23], the proposed method achieves the best results among the compared TMOs.

Since the main goal of this work is the application of tone mapping in an autonomous driving context, we have evaluated the effectiveness of several successful classical state-of-the-art tone mapping methods from the literature [9,24,25,26,27]. They were evaluated in terms of object detection performance applied to the tone-mapped outputs of each method. The evaluation confirmed our assumption that quality metrics such as SNR, contrast, or color saturation are not indicative of the performance of each tone mapping method in detection applications, and methods can vary in their robustness to scene conditions. Based on a comparison between many different state-of-the-art methods, we have selected the method of Farbman et al. [9] as a state-of-the-art representative in further evaluation. This is an edge-preserving filtering technique based on weighted least-squares optimization. In HDR tone mapping, it is used for compressing the luminance while preserving the details.

In the proposed method, all algorithm design choices consider the effect on the detection of traffic road users. For evaluation purposes, we relied on HDR images simulated from images in a public SDR traffic dataset [28], as well as our multi-modal traffic dataset including true HDR data with “person” annotations [29].

## 3. Proposed Method

In this paper, we present a tone mapping method that generalizes to the large variety of light conditions that is encountered in driving scenarios. The optimization of the algorithm is set in a learning-based framework using a convolutional neural network (CNN) trained to perform tone mapping of HDR images while maximizing image quality and object detection objectives.

The proposed method introduces three main novelties:

- The method emphasizes accuracy in image regions containing objects of interest by increasing the sampling density of these image regions during the neural network’s stochastic gradient descent training.
- During training, data augmentation techniques are used to improve the model’s robustness to challenging lighting conditions, particularly in traffic situations. The augmentations are informed by an analysis of factors identified as having a significant impact on detection performance in a study we conducted for this research.
- Unlike most HDR methods in the literature, the method is evaluated using real driving data, with detection accuracy measured in the context of traffic safety. Additionally, the method’s tone mapping performance is evaluated on challenging data, which is a small but important subset of traffic situations.

### 3.1. Network Architecture

The design of the network architecture was inspired by ExpandNet [21], which is a framework originally designed for HDR expansion (inverse tone mapping). In this work, we adapted a simplified architecture based on ExpandNet for tone mapping. The architecture utilizes multi-scale processing and draws from successful classical approaches while maintaining low complexity.

**Figure 1:**
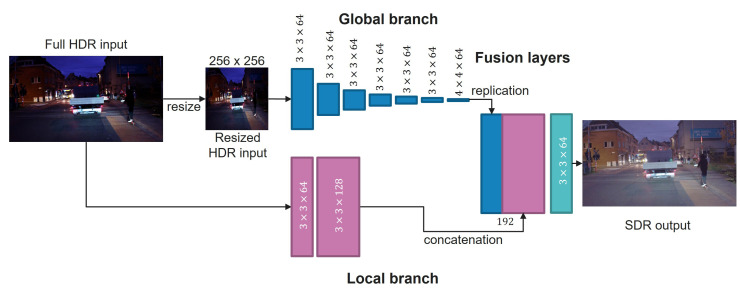
The proposed tone mapping architecture was inspired by ExpandNet [21], and it was simplified by discarding a branch of layers called the “dilation branch”, which operates on the full resolution with a wide perceptive field and is therefore computationally complex. The network is comprised of convolutional layers followed by ReLU activations. The global branch spatially down-samples the feature maps in each subsequent layer through skip convolutions, and the local branch operates at the original resolution. The fusion layers combine the local and global features into an output tone-mapped image.

Figure 1 illustrates the structure of the proposed tone mapping method. The full-resolution HDR image is passed through convolution layers of size 3×3×64 and convolutions of size 3×3×128, followed by non-linear activations (ReLUs) in a local processing branch. The local branch encodes local image features related to object edges and structures, with the purpose of ensuring correct contrast compression without contrast inversion and halo artifacts. A global branch applies strided 3×3×64 convolutions followed by ReLUs and a 4×4×64 convolution to summarize the global illumination into a single, 64-element vector. The effect of this branch can be interpreted as analogous to calculating a global image histogram in classical methods. A final fusion branch concatenates the local features with the global representation and applies two full-resolution convolution layers: a convolution of size 3×3×64 followed by a final layer of size 1×1×3. The output is a tone-mapped RGB image suitable for 8-bit quantization without a significant loss of details in the relevant luminance ranges and local regions.

Computational efficiency is an important constraint for computer vision systems in autonomous driving and advanced driver assistance systems (ADAS) applications. Considering efficiency, we identified the middle “dilation” branch from the original ExpandNet architecture as the least contributing and omitted it in the simplified design shown in Figure 1 while still preserving the local and global branches. The number of trainable parameters was reduced by approximately 25%, and our experiments confirmed that the reduction did not cause a noticeable loss in learning capability.

Consequently, the proposed tone mapping architecture has 340,227 trainable parameters, which is only 0.5% of the size of YOLO v3. In terms of calculations, our architecture performs 290 million floating-point operations (GFLOPs) for a 3-channel image of size 1.6 Mpixels, which is approximately 46% of the complexity of YOLO v3 for the same image size.

### 3.2. Training Cost Function

Our tone mapping CNN is trained to map the intensity range of the input HDR images by enforcing similarity between the output and a reference SDR image. The original similarity loss proposed in [21] is suitable in our framework as well since it ensures correct color and detail reconstruction. Following the definition in [21], the loss L(Z,Y) between the output image Z and the reference image Y is defined as a linear combination of the mean $L_{1}$-based loss and the mean cosine similarity-based loss $L_{\mathrm{CS}}$, with a constant linear coefficient λ=5:

(1) $$L\left(Z,Y\right)=L_{1}\left(Z,Y\right)+\lambda L_{\mathrm{CS}}\left(Z,Y\right),$$

where $L_{1}\left(Z,Y\right)$ is the mean absolute difference between the output image Z and the ground truth image Y averaged over all three RGB color channels and all *N* pixel locations. This loss was chosen due to its robustness to outliers and was found to result in sharper images compared to $L_{2}$-based loss.

The cosine similarity (CS) reflects a relative similarity between two colors and is invariant to the absolute intensity (the vector magnitude). It is calculated as the inner product of the vectors of RGB intensities at matching pixel locations in the two images, normalized by the product of their magnitudes (Equation (2)). The cosine similarity-based loss $L_{\mathrm{CS}}$ used in this work is inversely proportional to the cosine similarity metric:

(2) $$L_{\mathrm{CS}}\left(Z,Y\right)=1-\frac{1}{N}\sum_{i=1}^{N}\frac{\left[Z_{i,R},Z_{i,G},Z_{i,B}\right]\cdot {\left[Y_{i,R},Y_{i,G},Y_{i,B}\right]}^{T}}{∥[\left[Z_{i,R},Z_{i,G},Z_{i,B}\right]∥\;∥\left[Y_{i,R},Y_{i,G},Y_{i,B}\right]∥}.$$

In the HDR-to-SDR mapping context, this loss is suitable for ensuring correct color reconstruction, which is less sensitive to luminance compression in the tone mapping process.

### 3.3. Data Pre-Processing and Augmentation

Currently, to the best of our knowledge, there is no extensive, publicly available annotated traffic dataset of high-dynamic-range images. Therefore, we relied on a public automotive SDR dataset [28], based on which we created synthetic HDR training images. The modifications that simulate different challenging situations applied as an augmentation to the training set were also applied for controlled, simulated experiments for validation. Furthermore, for evaluation in real conditions, we collected and annotated our own dataset with true HDR images [29].

After investigating the main contributing factors that negatively affect detection performance, we focused on two aspects: the robustness to noise and the image brightness in dark scenes. Modifying the intensity values in dark areas can increase the prominence of noise and negatively impact the quality and thus detection performance. Our proposed contribution to mitigating this problem is to focus on improving the contrast-to-noise ratio of the output images, as described below.

**Figure 2:**
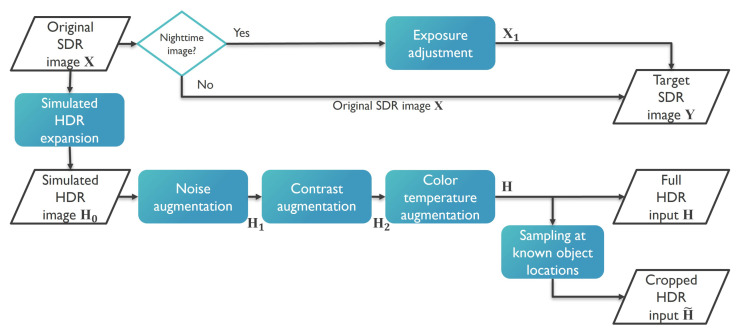
Flowchart of the process of creating synthetic HDR training data from SDR images. The blue blocks represent the data pre-processing steps that comprise the proposed detection-informed training procedure. It focuses on creating realistic and challenging training conditions. The training inputs that are created are an HDR input image and a crop from the same image centered at a known object location, and they are coupled with a training ground-truth (target) SDR image.

To create a training dataset, we applied different pre-processing and data augmentation steps to the original SDR images and obtained pairs of input HDR and ground truth SDR images. This process is illustrated in Figure 2 and summarized in the following steps:

1. A collection of SDR images $X_{1},X_{2},\cdots$ is used as a source for simulating input HDR images, as well as for a reference during training.
2. If a dark SDR training image is identified, its exposure is increased by simulating a higher sensor gain. In the case of a bright (daylight) image, the exposure remains unchanged. This becomes the target SDR ground truth image Y.
3. The original SDR image is converted into an HDR image $H_{0}$ using a dynamic range expansion operator [21]. Poisson noise is added with a random noise variance, through mosaicking/demosaicking ($H_{1}$).
4. A contrast remapping is performed to simulate (augment) alternative and more challenging light conditions in terms of light intensity contrast ($H_{2}$).
5. To make the model robust to various light sources and daylight conditions, a slight color temperature shift is applied to each training image randomly. This results in the final simulated HDR image (H). The full HDR image is used as an input into the global branch of the network responsible for global illumination compression.
6. Lastly, to give higher significance to the reconstruction of objects of interest, a region is cropped from the HDR image which is sampled around the center of a known object location. This region ($\tilde{H}$) is the input to the local branch of the network responsible for the local contrast mapping.

#### 3.3.1. Datasets

The Berkeley Deep Drive (BDD) dataset [28] consists of diverse images of daylight, night-time, and dawn/dusk traffic scenes in urban scenarios. A collection of corresponding object annotations are available in the form of 2D bounding box coordinates and labels for multiple road user categories. The images in the dataset are captured using the automatic exposure settings of an SDR camera, and stored in an 8-bit representation.

For training we used 3657 images from the BDD dataset. The validation set consists of a different set of 550 images used to evaluate the model during training, and another 99 images were selected as a test set for objective evaluation of the algorithm.

Our collection of HDR test data [29] is part of a multi-modal dataset recorded in traffic conditions and annotated in a semi-automatic fashion with labels of the class “person”, including pedestrians and cyclists. In this work, we used an actual high-dynamic-range camera (using the Sony IMX490 sensor) with a dynamic range of 120 dB. The image data were saved in a 24-bit format. We used approximately 370 diverse test images captured in daylight, twilight, and at night.

#### 3.3.2. Enhancement of Dark Ground Truth Training Images

Our small-scale investigation revealed that in the majority of dynamic range conditions, object detectors are robust to a decrease in contrast. However, low contrast negatively impacts the detection confidence, and from a picture quality perspective in a driver assistance context, low contrast is also undesirable. Therefore, the proposed approach increases the brightness of nighttime ground truth images to train the network to enhance the visibility of objects. Specifically, a nighttime target SDR image Y at pixel location *i* is created by increasing the exposure of the original nighttime SDR image $X_{1}$ by s∈[0.5,1.5] stops as:

(3) $$Y_{i}={\left(X_{1,i}^{\beta }\cdot 2^{s}\right)}^{1/\beta },$$

where the exponential mapping with β=2.2 serves as a replacement of a camera response curve, and the parameter *s* is determined by random sampling from a uniform distribution to increase the variability in the data and the robustness of the model.

#### 3.3.3. Noise Augmentation

**Figure 3:**
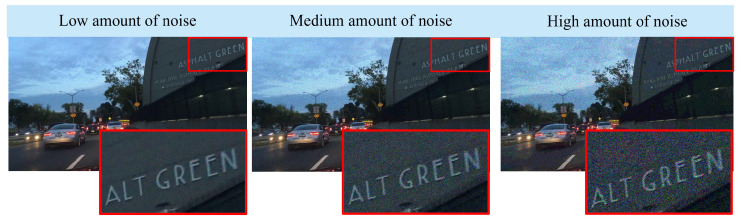
Example of simulating different amounts of Poisson noise to augment the training set and create a model robust to noise.

Since noise is inextricably linked to low-light conditions, we propose to incorporate a noise-augmented training procedure. Noise robustness is achieved by augmenting the training dataset with noisy images by adding different levels of signal-dependent Poisson noise in Bayer mosaicked (raw) image data. The parameters of the Poisson noise distribution are: k=1, and λ is randomly sampled from a range between 0.2% of the pixel intensity $H_{0,i}$ (corresponding to low amount of noise) and 20% of the pixel intensity $H_{0,i}$ (high amount of noise). The images are then demosaicked using bilinear interpolation into full-resolution color images before providing them as input to the neural network. The mosaicking/demosaicking aspect is important because it simulates a real sensor and because this process affects the spatial correlation of the noise in neighboring pixels and therefore the local contrast. An example of the results of this procedure applied to an SDR image for visualization purposes is shown in Figure 3.

#### 3.3.4. Contrast Augmentation Procedures

The dynamic range of real-world scenes can vary depending on light sources and atmospheric conditions. For example, a sunlit daytime urban scene is typically about five orders of magnitude brighter than an artificially illuminated street at night [20]. Similarly, the contrast between dark areas and bright lights in nighttime scenes can have a ratio of $10^{4}:1$.

One of the contributions of this paper is the data augmentation approach focusing on contrast robustness. By simulating realistic conditions, a pool of contrast mapping techniques is created, including gamma encoding, sigmoidal contrast stretching, and selective region-based non-linear mapping described further. At each iteration, for each training image, one contrast mapping function is randomly selected and applied to the simulated HDR output form ExpandNet [21].

Gamma encoding performs non-linear mapping of the image intensity values $H_{1,i}$ normalized in the range [0, 1]: $H_{2,i}=H_{1,i}^{\gamma }$. In our augmentation procedure, we applied gamma expansion with γ∈[1,3], selected by random sampling for each training image, to further expand the dynamic range and effectively increase the contrast by making the shadows darker. This procedure simulates variability in ambient light in the environment.

The sigmoidal contrast stretching maps the normalized luminance range into a sigmoidal shape as $H_{2,i}=\frac{H_{1,i}}{1+e^{-kH_{1,i}}}$, thus stretching the difference between the dark and the bright areas within the same bit-depth [30]. By varying k∈[4,12], which determines the steepness of the sigmoid, various dynamic range versions of the scene are created at each training iteration, simulating a sensor with more limited dynamic range capabilities. With steeper curves, the dark areas become darker and close to the lower sensitivity threshold, while the bright areas become closer to the saturation limit.

For simulating even more challenging situations in the scene, we applied a selective degradation procedure that selectively suppresses the luminance in the shadows, without significantly disturbing the bright areas. A selective mask S was used to identify the shadow regions in the image. Given an input HDR image $H_{1}$ with intensity normalized in the range [0,1], the selective mask at pixel *i* is defined as $S_{i}=\frac{1}{1+e^{-20(H_{1,i}-t)}}$. The threshold *t* to select between shadows and bright areas is determined by uniform random sampling between 60% and 70% of the maximum possible luminance intensity t∈[0.6,0.7]. Finally, the simulation of challenging conditions is achieved as a linear combination as $H_{2,i}=\left(1-S_{i}\right)cH_{1,i}+S_{i}H_{1,i}$, where $cH_{1,i}$ is a luminance-compressed (dark) representation of the image $H_{1}$ at pixel *i*, using an experimentally chosen parameter c=0.004. Such modifications correspond to challenging situations such as entering a tunnel or a camera blinded by strong direct light. Easy conditions are simulated by selecting parameters that do not increase the contrast significantly, and medium (mixed) conditions are obtained by blending an easy and a challenging version of a frame, using a random blending weight.

#### 3.3.5. Color Temperature Change

The motivation to augment the dataset with a variable color temperature results from the observation that the ambient light throughout the day has a variable color temperature and that camera compensation for it is often inadequate or absent, especially in those challenging conditions. Taking the color temperature into consideration assists the process of learning to generalize to images of different color balances. In the augmentation process, we simulate color temperature variations from 2000 K to 10,000 K following [31]. Since state-of-the-art object detectors are highly robust to the color balance, this augmentation predominantly contributes to the visual quality of the image and only affects detection performance in corner cases which significantly distort the natural appearance of the colors.

### 3.4. General Training Details

**Figure 4:**
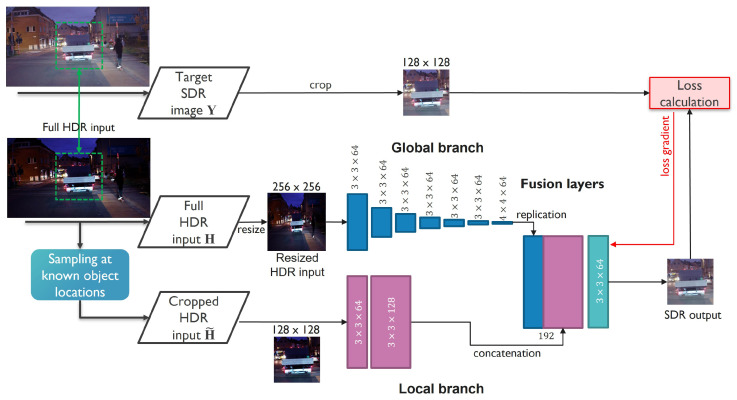
An illustration of the proposed training approach using crops at object-centered locations to focus on reconstruction of details. The size of the convolution kernels is indicated by the numbers at the corresponding feature maps of each layer.

As illustrated in Figure 4, the global branch summarizes the entire image into a single 64-element vector through strided convolutions. Due to the large dimensionality reduction in this branch, preservation of fine image details is not essential. Therefore, for efficiency, the image is resized to a fixed size of 256×256 pixels before being processed by the global branch.

The local branch looks at local image patches at each pixel location and requires full-resolution image information, without any loss of detail. To achieve a memory- and computationally-efficient method, this is implemented by cropping and processing rectangular regions taken from the full-resolution image. To that end, another contribution of this paper is the selection of regions for training that are fed into the local branch. Since the end goal is the application of the proposed method in object detection in traffic scenes, the quality of the output has the highest importance in regions with important road users. With the aforementioned considerations, for training, we used crops of size 128×128 pixels extracted through weighted random sampling, focusing on areas where the presence of at least one object is known from the ground-truth labels.

The network was trained using the Adam [32] optimizer, with an initial learning rate of $10^{-3}$, reduced to $10^{-4}$ during the final epochs, for approximately 3500 epochs, each with a random variation of the original training set. The batch size was gradually increased to 16. Training a lightweight neural network using the Adam optimizer combined with extensive data augmentation helps to mitigate a common training-time issue of vanishing gradients [33].

The proposed method was developed in the Python programming language using the PyTorch framework on a Linux operating system. The CNN training and inference processing was carried out and tested using an Nvidia GeForce RTX 2080 Ti graphics card with 11 GB of memory.

## 4. Experiments and Results

To evaluate the contributions of the proposed training procedure, we carried out an ablation study, where initially a baseline model was trained without the proposed novelties: the ground truth images were the original (non-enhanced) SDR images, no noise was added, and the region crops were sampled at uniformly distributed random positions as inputs to the local branch. The baseline model is used as a reference for comparison with our proposed training strategy that is informed by an empirical analysis of factors related to detection performance.

The quality of the tone mapping output was evaluated based on the object detection performance of the YOLO v3 object detector [34]. We used a YOLO model pre-trained on the COCO dataset [35], and considered only the traffic-related objects from its multi-class output.

For a comprehensive analysis, we evaluated the results in several aspects: robustness to challenging light conditions, robustness to noise, and overall detection performance by comparison with the state-of-the-art in tone mapping. After a rigorous comparison evaluation study, the algorithm by Farbman et al. [9] was selected as a representative of the state-of-the-art (SOTA), due to its strong performance in various conditions.

### 4.1. Evaluation with Simulated HDR Data

In the first set of experiments, the goal is to evaluate the contribution of the proposed novelties towards improving the detection quality. The comparison with the baseline model is to assess relative improvement by applying informed training and comparing it to the selected classical state-of-the-art method illustrates the absolute performance of the proposed method.

The first set of experiments focuses on evaluating the robustness of the proposed method to dynamic range (DR) conditions in the scene. To this end, three different versions of the test set were created: non-challenging, medium, and challenging set. More specifically, from the available SDR images in the Berkeley dataset [28], HDR images were simulated by dynamic range expansion [21] and applying the selective degradation procedure described in Section 3.3.4 with increasing degradation levels.

The results in Table 1 demonstrate that the proposed tone mapping method DI-TM significantly outperforms that of Farbman et al. [9] in the challenging cases. We reason that the strength of the proposed network lies in the training on a versatile set of realistic conditions specific to traffic situations, such as blinding headlights or street lights at night and direct sunlight also reflecting from the road. The two methods perform similarly well on the medium and the non-challenging sets, showing that our model is robust to a range of different illumination levels while mitigating the need for initial parameter adaptation. In this experiment, the comparison with our baseline model verifies that the proposed contrast augmentation steps are effective for training a robust model.

**Figure 5:**
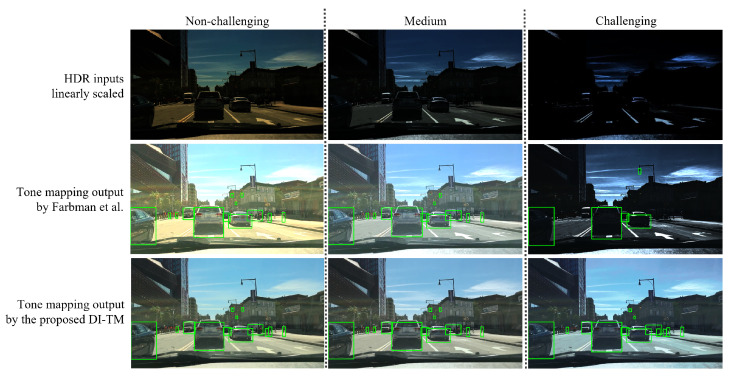
Example of the performance of the reference SOTA tone mapping method Farbman et al. [9] and the proposed DI-TM in variable scene dynamic range conditions. The green bounding boxes represent correct detection outputs (true positives) for “person”, “car”, and “traffic light” combined. The proposed method DI-TM is more robust in variable and extreme contrast conditions.

As an illustration, Figure 5 provides an example of the different levels of simulated dynamic range conditions and shows the output of the comparison with the method of Farbman et al. [9]. The proposed DI-TM method produces more consistent tone mapping results across a range of challenging conditions thanks to the robust training strategy. Furthermore, in the output of the proposed method, more objects are correctly detected in challenging conditions due to better detail preservation and contrast adaptation.

From a qualitative perspective, we evaluated the proposed method in terms of its robustness to noise. In this experiment, three variants of the test set were obtained by increasing the Poisson noise strength (see Figure 3) that is added to the HDR test set. The results in Table 2 indicate that, due to the denoising properties of the proposed method, object detection is less affected by noise in all three categories compared to the method of Farbman et al. [9]. Furthermore, compared to the baseline model, the noise augmentation approach proves to be beneficial for detection since it helps the network learn to generate less noisy images.

### 4.2. Evaluation with True HDR Data

To assess the performance in real-world conditions, we relied on a diverse subset of 372 annotated frames of our own captured dataset using a pair of SDR and HDR cameras, aligned and synchronized to match their viewpoints. In total, this subset contains 1398 “person” objects. Comparing to SDR helps to evaluate the benefits of using an HDR sensor in the automotive context. For objective evaluation of the proposed method, we relied on the comparison with the method of Farbman et al. [9].

The first experiment focused on the difficulty of the light conditions in terms of dynamic range, similar to the experiment in Section 4.1, where high-contrast scenes are considered more challenging for visibility than low-contrast scenes. To keep the original pixel intensity distribution unchanged, the distinction between easy and challenging subsets was achieved by manually identifying images of low and high contrast. Due to its higher representation in the dataset as well as the object detection reliability, this experiment evaluated only the category “person”.

In Table 3, the detection performance of the object detector for the class “person” is presented for easy and challenging dynamic range conditions. The results confirm that using HDR data is beneficial, especially in challenging conditions. Furthermore, the results show that adapting the tone mapping method specifically for object detection in the automotive context brings an additional contribution to the detection quality. We believe that this is a result of the extensive data augmentation process combining variable contrast, illumination, and noise simulation. Such conditions are more scarce in typical driving conditions; however, they are critical for the safety and reliability of intelligent vehicles.

**Figure 6:**
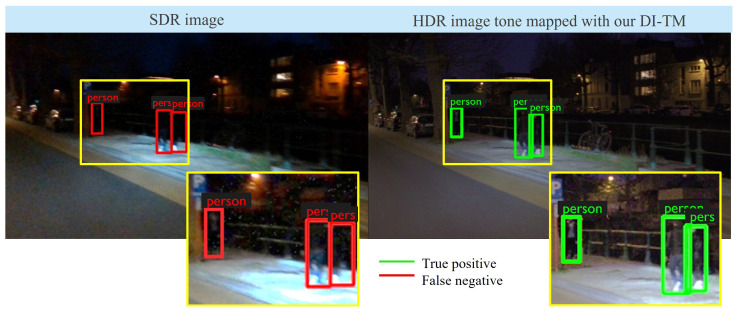
An example of a challenging scene in our dataset of SDR and true HDR images. In an SDR representation, much of the contrast at the object edges is lost, and the objects in the darkness are invisible to the detector. HDR images preserve fine intensity differences, and our tone mapping method enhances the details such that they become visible for the detector, as well as for visualization to a human driver.

An example of a challenging night-time scene is presented in Figure 6. The cropped regions were further manually enhanced only for visualization purposes. It can be noticed that due to the high contrast, the pedestrians in the darkness were missed in the SDR image, while they were detected in the tone-mapped output using the proposed DI-TM. Due to the highly variable augmentation, our method is robust to changing scene conditions without any manual interventions, unlike the method of Farbman et al. [9]

**Figure 7:**
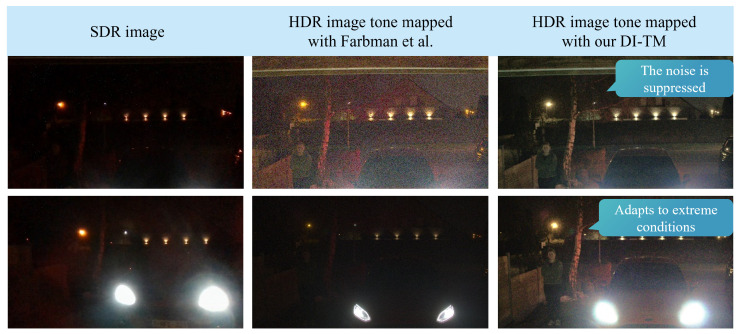
Qualitative evaluation of the robustness of Farbman et al. [9] vs. proposed model DI-TM1 in extremely challenging high-contrast night-time scenes.

Finally, as a qualitative evaluation, we present an experiment focusing on extreme dynamic range conditions. In this evaluation, the model was applied to samples from our recorded true HDR dataset. Examples of this evaluation are presented in Figure 7. The proposed method can suppress the noise, and it is more robust to variable and highly challenging conditions compared to the representative of the classical state-of-the-art.

Both the qualitative and the quantitative evaluations confirm the effectiveness of the image pre-processing strategies involved in data augmentation. The ideas were motivated by realistic driving conditions and observations following an investigation into the performance of object detectors in various high-contrast conditions. On the other hand, the method of Farbman et al. [9] is among the state-of-the-art in classical tone mapping algorithms; however, it is optimized for aesthetic aspects of the image quality in photography and cinematic applications.

## 5. Conclusions

In this study, our focus lies in the design of an HDR tone mapping model aimed at enhancing image quality while considering the objectives of object detection. We believe that this approach holds significant potential for facilitating the integration of HDR cameras into ADAS and autonomous vehicle perception systems. We investigated different factors contributing towards good-quality tone mapping, informed by the performance of object detectors on the tone mapping outputs.

We proposed a detection-informed strategy for tone mapping, referred to as DI-TM, and evaluated its performance in multiple aspects, including image quality and detection accuracy. Our findings demonstrate that the proposed approach outperforms classical state-of-the-art tone mappers in easy and challenging lighting conditions while maintaining robustness across different noise levels. Compared to using an SDR sensor, the detection performance improvement in challenging light conditions was 49% as measured by the $F_{2}$ score, which highlights the advantage of integrating HDR cameras in intelligent vehicles. Compared to a state-of-the-art tone mapping method optimized for visual quality, with our detection-optimized approach, we achieved an improvement of 13% in detection accuracy.

These results suggest the promising potential for informed, task-based algorithm design improvements in the already very researched field of HDR tone mapping, with future applications in enriching the vision systems of autonomous driving platforms.

Future research will focus on exploring the robustness of the proposed training approach in multiple-vehicle perception tasks, examining its effectiveness across various scenarios. Additionally, there is a need to explore the tradeoff between computational efficiency and performance, especially in the context of adapting existing computer vision tools or developing novel techniques tailored to handle high-dynamic-range (HDR) data. This investigation will contribute to the optimization and advancement of more accurate environment perception systems by application of HDR data in the automotive domain.

## Figures and Tables

**Table 1:** Object detection performance (average F2 score for “person”, “car”, and “traffic light”) of YOLO v3 [34] applied to the outputs of the classical-state-of-the-art method of Farbman et al. [9] on our baseline model and the outputs from the proposed detection-informed method DI-TM in scenes of different dynamic range. The bold numbers indicate the best performance among the listed methods in each category.

	Average F2 Score per Scene Type		
**Method**	**Non-Challenging**	**Medium**	**Challenging**
Farbman et. al [9]	0.48	0.48	0.32
Baseline model	0.49	0.46	0.37
Proposed DI-TM	**0.50**	**0.49**	**0.44**

**Table 2:** Object detection performance of YOLO v3 [34] on the outputs of the classical-state-of-the-art method of Farbman et al. [9], our baseline model and the proposed detection-informed method DI-TM in variable degrees of Poisson noise. The bold numbers indicate the best performance among the listed methods in each category.

	Average F2 Score per Noise Level		
**Method**	**Low**	**Medium**	**High**
Farbman et. al [9]	0.43	0.35	0.22
Baseline model	0.43	0.36	0.26
Proposed DI-TM	**0.45**	**0.40**	**0.34**

**Table 3:** Object detection performance of YOLO v3 [34] on matching SDR frames, on the outputs of Farbman et al. [9], and on the proposed detection-informed method DI-TM in selected subsets of easy (lower dynamic range) and hard (high dynamic range) examples from our own collected dataset for the category “person”. The bold numbers indicate the best performance among the listed methods in each category.

	F2 Score per Scene Type	
**Method**	**Easy**	**Challenging**
SDR data	0.68	0.41
Farbman et al. [9]	0.73	0.54
Proposed DI-TM	**0.75**	**0.61**

## Data Availability

The data presented in this study are available on request from the corresponding author. The data are not publicly available due to privacy restrictions.

## References

[1] Macek K. (2022). Pedestrian Traffic Fatalities by State: 2021 Preliminary Data.

[2] NHTSA’s National Center for Statistics and Analysis (2019). Pedestrians: 2017 Data. Traffic Safety Facts Report No. DOT HS 812 681.

[3] Teoh E.R., Kidd D.G. (2017). Rage against the machine? Google’s self-driving cars versus human drivers. J. Saf. Res..

[4] Kalra N., Paddock S.M. (2016). Driving to safety: How many miles of driving would it take to demonstrate autonomous vehicle reliability?. Transp. Res. Part A Policy Pract..

[5] Di X., Shi R. (2021). A survey on autonomous vehicle control in the era of mixed-autonomy: From physics-based to AI-guided driving policy learning. Transp. Res. Part C Emerg. Technol..

[6] Commission E. (2022). New Rules to Improve Road Safety and Enable Fully Driverless Vehicles in the EU. https://ec.europa.eu/commission/presscorner/detail/en/IP_22_4312.

[7] Reinhard E., Stark M., Shirley P., Ferwerda J. Photographic tone reproduction for digital images. Proceedings of the 29th Annual Conference on Computer Graphics and Interactive Techniques.

[8] Kuang J., Johnson G.M., Fairchild M.D. (2007). iCAM06: A refined image appearance model for HDR image rendering. J. Vis. Commun. Image Represent..

[9] Farbman Z., Fattal R., Lischinski D., Szeliski R. (2008). Edge-preserving decompositions for multi-scale tone and detail manipulation. ACM Trans. Graph. (TOG).

[10] Li Y., Liao N., Wu W., Deng C., Li Y., Fan Q., Liu C. (2023). Tone Mapping Operator for High Dynamic Range Images Based on Modified iCAM06. Sensors.

[11] Goswami A., Ak A., Hauser W., Le Callet P., Dufaux F. Reliability of Crowdsourcing for Subjective Quality Evaluation of Tone Mapping Operators. Proceedings of the 2021 IEEE 23rd International Workshop on Multimedia Signal Processing (MMSP).

[12] Cerdá-Company X., Párraga C.A., Otazu X. (2018). Which tone-mapping operator is the best? A comparative study of perceptual quality. J. Opt. Soc. Am. A.

[13] Su C.C., Wang R., Lin H.J., Liu Y.L., Chen C.P., Chang Y.L., Pei S.C. Explorable tone mapping operators. Proceedings of the 2020 25th International Conference on Pattern Recognition (ICPR).

[14] Rana A., Singh P., Valenzise G., Dufaux F., Komodakis N., Smolic A. (2019). Deep tone mapping operator for high dynamic range images. IEEE Trans. Image Process..

[15] Panetta K., Kezebou L., Oludare V., Agaian S., Xia Z. (2021). Tmo-net: A parameter-free tone mapping operator using generative adversarial network, and performance benchmarking on large scale hdr dataset. IEEE Access.

[16] Wang C., Chen B., Seidel H.P., Myszkowski K., Serrano A. (2022). Learning a self-supervised tone mapping operator via feature contrast masking loss. Proceedings of the Computer Graphics Forum.

[17] Zhang J., Wang Y., Tohidypour H., Pourazad M.T., Nasiopoulos P. A Generative Adversarial Network Based Tone Mapping Operator for 4K HDR Images. Proceedings of the 2023 International Conference on Computing, Networking and Communications (ICNC).

[18] Mukherjee R., Melo M., Filipe V., Chalmers A., Bessa M. (2020). Backward compatible object detection using hdr image content. IEEE Access.

[19] Mukherjee R., Bessa M., Melo-Pinto P., Chalmers A. (2021). Object detection under challenging lighting conditions using high dynamic range imagery. IEEE Access.

[20] Onzon E., Mannan F., Heide F. Neural auto-exposure for high-dynamic range object detection. Proceedings of the IEEE/CVF Conference on Computer Vision and Pattern Recognition.

[21] Marnerides D., Bashford-Rogers T., Hatchett J., Debattista K. (2018). Expandnet: A deep convolutional neural network for high dynamic range expansion from low dynamic range content. Proceedings of the Computer Graphics Forum.

[22] Koçdemir İ.H., Koz A., Akyuz A.O., Chalmers A., Alatan A., Kalkan S. (2022). Tmo-Det: Deep Tone-Mapping Optimized with and for Object Detection. https://papers.ssrn.com/sol3/papers.cfm?abstract_id=4132028.

[23] Yeganeh H., Wang Z. (2012). Objective quality assessment of tone-mapped images. IEEE Trans. Image Process..

[24] Reinhard E., Devlin K. (2005). Dynamic range reduction inspired by photoreceptor physiology. IEEE Trans. Vis. Comput. Graph..

[25] Mantiuk R., Myszkowski K., Seidel H.P. (2006). A perceptual framework for contrast processing of high dynamic range images. ACM Trans. Appl. Percept. (TAP).

[26] Drago F., Myszkowski K., Annen T., Chiba N. (2003). Adaptive logarithmic mapping for displaying high contrast scenes. Proceedings of the Computer Graphics Forum.

[27] Durand F., Dorsey J. Fast bilateral filtering for the display of high-dynamic-range images. Proceedings of the 29th Annual Conference on Computer Graphics and Interactive Techniques.

[28] Yu F., Chen H., Wang X., Xian W., Chen Y., Liu F., Madhavan V., Darrell T. Bdd100k: A diverse driving dataset for heterogeneous multitask learning. Proceedings of the IEEE/CVF Conference on Computer Vision and Pattern Recognition.

[29] Dimitrievski M., Shopovska I., Van Hamme D., Veelaert P., Philips W. Automatic labeling of vulnerable road users in multi-sensor data. Proceedings of the 2021 IEEE International Intelligent Transportation Systems Conference (ITSC).

[30] Saruchi S. (2012). Adaptive sigmoid function to enhance low contrast images. Int. J. Comput. Appl..

[31] Helland T. (2012). How to Convert Temperature (K) to RGB: Algorithm and Sample Code. https://tannerhelland.com/2012/09/18/convert-temperature-rgb-algorithm-code.html.

[32] Kingma D.P., Ba J. (2014). Adam: A method for stochastic optimization. arXiv.

[33] Abuqaddom I., Mahafzah B.A., Faris H. (2021). Oriented stochastic loss descent algorithm to train very deep multi-layer neural networks without vanishing gradients. Knowl. Based Syst..

[34] Redmon J., Farhadi A. (2018). Yolov3: An incremental improvement. arXiv.

[35] Lin T.Y., Maire M., Belongie S., Hays J., Perona P., Ramanan D., Dollár P., Zitnick C.L. Microsoft coco: Common objects in context. Proceedings of the 13th European Conference on Computer Vision.

